# Multi-omic interactions in the gut of children at the onset of islet autoimmunity

**DOI:** 10.1186/s40168-022-01425-6

**Published:** 2022-12-16

**Authors:** Patrick G. Gavin, Ki Wook Kim, Maria E. Craig, Michelle M. Hill, Emma E. Hamilton-Williams

**Affiliations:** 1 Frazer Institute, The University of Queensland, Woolloongabba, QLD Australia; 2 Present Address: Pulmonary and Critical Care Medicine, Brigham and Women’s Hospital, Boston, MA USA; 3 Present Address: Harvard Medical School, Boston, MA USA; 4 Virology Research Laboratory, Prince of Wales Hospital Randwick, Sydney, Australia; 5 School of Clinical Medicine, Discipline of Paediatrics and Child Health, Faculty of Medicine and Health, University of New South Wales, Sydney, Australia; 6 Institute of Endocrinology and Diabetes, Children’s Hospital at Westmead, Sydney, Australia; 7 Discipline of Child and Adolescent Health, University of Sydney, Sydney, Australia; 8 QIMR Berghofer Medical Research Institute, Brisbane, QLD Australia

**Keywords:** Gut microbiota, Microbiome, Metaproteome, Virome, Type 1 diabetes, Islet autoimmunity, Infant/child, *Faecalibacterium*, Mastadenovirus

## Abstract

**Background:**

The gastrointestinal ecosystem is a highly complex environment with a profound influence on human health. Inflammation in the gut, linked to an altered gut microbiome, has been associated with the development of multiple human conditions including type 1 diabetes (T1D). Viruses infecting the gastrointestinal tract, especially enteroviruses, are also thought to play an important role in T1D pathogenesis possibly via overlapping mechanisms. However, it is not known whether the microbiome and virome act together or which risk factor may be of greater importance at the time when islet autoimmunity is initiated.

**Results:**

Here, we apply an integrative approach to combine comprehensive fecal virome, microbiome, and metaproteome data sampled before and at the onset of islet autoimmunity in 40 children at increased risk of T1D. We show strong age-related effects, with microbial and metaproteome diversity increasing with age while host antibody number and abundance declined with age. Mastadenovirus, which has been associated with a reduced risk of T1D, was associated with profound changes in the metaproteome indicating a functional shift in the microbiota. Multi-omic factor analysis modeling revealed a cluster of proteins associated with carbohydrate transport from the genus *Faecalibacterium* were associated with islet autoimmunity.

**Conclusions:**

These findings demonstrate the interrelatedness of the gut microbiota, metaproteome and virome in young children. We show a functional remodeling of the gut microbiota accompanies both islet autoimmunity and viral infection with a switch in function in *Faecalibacterium* occurring at the onset of islet autoimmunity.

Video Abstract

**Supplementary Information:**

The online version contains supplementary material available at 10.1186/s40168-022-01425-6.

## Background

Type 1 diabetes (T1D) is an autoimmune disease caused by immune cell destruction of the insulin-producing beta cells in the pancreatic islets. As with many autoimmune and auto-inflammatory disorders, the incidence of T1D has been steadily increasing for the past 50 years particularly in Western countries [1]. This increase has been too rapid to be caused by a change in genetic risk profile, but rather is thought to be caused by altered exposure to environmental risk factors [2]. The increase has been greatest in younger children and in those carrying low-risk HLA haplotypes suggesting altered gene-environmental interactions in early-life [3]. Multiple environmental risk factors have been proposed with many related to early-life diet (including breastfeeding and timing and type of solid food introduction), childhood obesity and infections, particularly from enteroviruses [2, 4, 5]. Together, these risk factors strongly suggest an involvement of the gut and the gut microbiota, with altered gut bacterial composition (dysbiosis), increased intestinal permeability and intestinal immune activation all reported to precede T1D diagnosis [6–9]. However, which environmental risk factors are of greater importance for the development of islet autoimmunity or whether they act in consort and how they may interact with each other to modify the host intestinal environment remains unknown.

While associations between T1D risk and the gut microbiota have tended to demonstrate variable findings between studies [10], several commonalities have been found. These include an increase in members of the genus *Bacteroides* associated with higher T1D risk and a decrease in taxa that produce short-chain fatty acids (SCFA), which are microbial metabolites produced from fermentation of dietary fiber [6, 7, 11–13]. SCFA have diverse benefits to the host with a role in promoting the integrity of the intestinal barrier, regulating appetite, suppressing inflammation and stimulating differentiation of regulatory T cells [10]. Taxa that produce high levels of the SCFA butyrate including genera *Clostridium*, *Eubacterium*, *Faecalibacterium*, *Roseburia*, and *Ruminococcus* and are generally considered beneficial for human health [10, 14]. However, the various studies in T1D tend to differ in which SCFA producing bacteria are reduced in abundance if at all [15]. One explanation for this is that the dysbiosis associated with T1D is functional rather than associated with specific taxa. In this study we incorporate metaproteomics along with traditional sequencing analysis to identify functional changes in the activity of the microbiota and gut together with taxonomic changes associated with the development of islet autoimmunity and the presence of viral infections.

Enterovirus infections are one of the most studied environmental factors associated with the presence of islet autoimmunity and T1D [4, 16]. Belonging to the *Picornaviridae* family, enteroviruses are ubiquitous single-stranded RNA viruses that are commonly transmitted through the fecal-oral route in children. Most enteroviruses establish primary infection and replication in the intestine but can ascend into the pancreas, where they can establish persistent infection [17, 18]. Longitudinal analysis of the gut virome (both mammalian and bacterial derived viruses) from children that developed islet autoimmunity and/or T1D found that prolonged enterovirus B shedding was associated with islet autoimmunity [19]. Furthermore, we previously showed that children with islet autoimmunity exhibit a greater abundance of enterovirus A species in the gut compared to islet autoantibody negative controls [20]. Mastadenoviruses of the *Adenoviridae* family are also common causes of human respiratory and gastrointestinal infection in childhood [21]. Children with islet autoimmunity exhibit fewer mastadenovirus C infections compared to controls, suggesting a possible protective effect of this virus, while human mastadenovirus F showed a weak positive correlation with the timing of seroconversion [19]. However, very little is known about possible mechanisms by which mastadenoviruses may impact islet autoimmunity or how any of these infections relate to the gut microbiota or intestinal barrier function.

Clinical onset of T1D is preceded by a period of ongoing islet autoimmunity detected by the presence of islet-specific autoantibodies (IAb) [22]. As seroconversion to IAb positivity is the first sign that an islet-specific immune response has been initiated, this is a likely time of action for a putative environmental trigger. However, environmental drivers may also act during the period between the appearance of IAb and clinical onset to accelerate disease progression. While multiple studies have examined associations between individual environmental factors such as the gut microbiota or viral infections with islet autoimmunity, there is a lack of integrated studies investigating interactions between these factors. We hypothesized that gastrointestinal viral infection may remodel the gut microbiota toward either a more dysbiotic state or a more beneficial phenotype depending on the type of infection. To test this hypothesis, we performed an exploratory multi-omic analysis using a cohort of children with stool samples collected before and at the time of seroconversion to islet autoimmunity, to examine the relationship between the gut microbiome, infection with vertebrate-infecting viruses and the stool metaproteome in children at-risk of T1D.

## Results

### Characterization of the gut microbiota, virome, and metaproteome before and after the onset of islet autoimmunity—study design

Participants included *n* = 40 children (20 cases who developed persistent IAb and 20 age-, sex-, and HLA-matched controls; Supplementary Table S1) from the Australian Viruses in the Genetically at Risk (VIGR) prospective birth cohort, a longitudinal observational study of children with a first-degree relative with T1D [20, 23]. Stool samples (*n* = 64) were collected from the cases at the time of seroconversion to islet autoimmunity and/or 15 ± 6 months prior. These stool samples were used for microbial community profiling via 16S rRNA sequencing and shotgun metaproteomics to assess functional characteristics of both the gut microbiota and host intestinal environment. These data were integrated with virome sequencing data of known vertebrate infecting viruses (excluding bacteriophages) from the same samples that was previously reported [20].

### Microbial diversity and abundance are associated with age

**Fig. 1:**
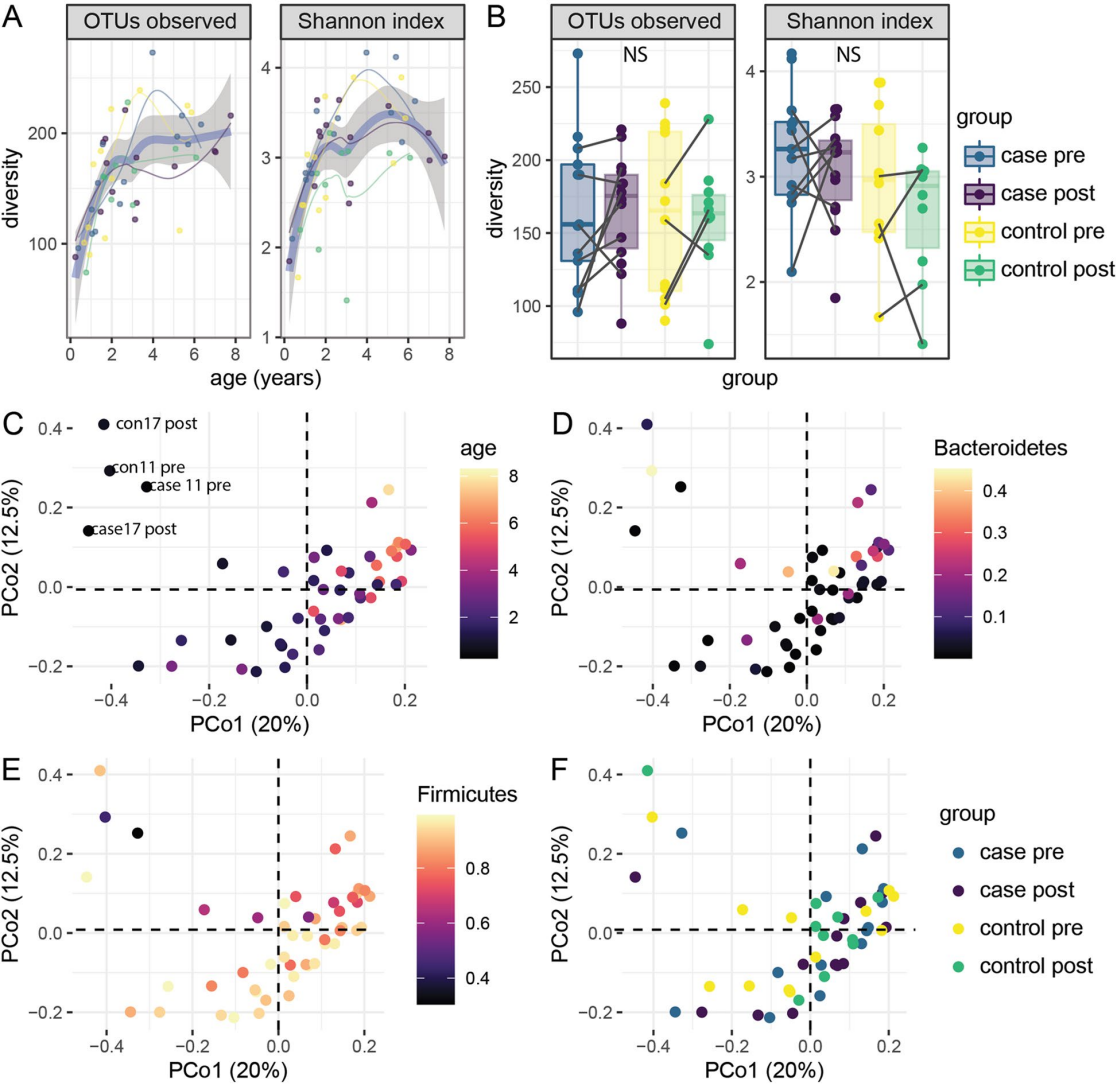
Microbial diversity and abundance are associated with age. The number of OTUs observed and Shannon index related to **A** age and **B** case and control status and timepoint. The thick blue curve represents all samples, the grey shaded are represents the 95% confidence interval, and the thinner curves represent the sample group. The first two principal coordinates of the Bray-Curtis distance are shown with each sample colored according to **C** age, **D** the proportion of counts attributed to Bacteroidetes, **E** the proportion of counts attributed to Firmicutes and **F** case group. Four samples (aged < 1 year of age) with a distinct microbiota are labeled in **C**. Case pre: IAb+ children prior to seroconversion, case post: IAb+ children after seroconversion, control pre: IAb− controls at first timepoint, control post: IAb− at second timepoint

As well documented [24], the richness, as measured by the number of observed OTUs, and the evenness, as measured using the Shannon index of the stool microbiome, both increased steadily during early childhood (Fig. 1A). In contrast to some previous reports [25, 26], case and control samples did not significantly differ in their alpha diversity (Fig. 1B, *p* = 0.85 for observed OTUs and *p* = 0.11 for Shannon). PCoA was used to investigate major drivers of variation in the stool microbiome. The dominant principal coordinates of the Bray-Curtis distances identified strong effects of age and the proportion of Bacteroidetes and Firmicutes on the first principal coordinate (Fig. 1C–E). Four samples from two age-matched pairs appear to have a very distinct microbiome composition (con11, con 17, case11, case 17, Fig. 1C). These samples were collected before 0.8 years of age and their unique composition may reflect the consumption of breast milk [27, 28]. These four younger aged samples also had lower diversity, with an average of 90 detected OTUs in samples taken in the first year versus 174 in the other samples. No grouping according to case-control status was observed within the first five principal coordinates, which collectively account for 53% of the variance (PCo1 and PCo2 are shown in Fig. 1F). Eighteen individual OTUs were significantly associated with the age of sample collection (Supplementary Table S2). These included three unclassified *Bacteroides* and 8 *Clostridiales*, consisting of 4 *Ruminococcaceae*, and 5 *Lachnospiraceae*, which increased with age (*q* < 0.1). A single OTU from *Erysipelotrichaceae* was found to decrease with age. These findings are consistent with previous reports that have found an increase in the proportion of Firmicutes after the adoption of solid food during early childhood as well as a drop in *Erysipelotrichaceae* after the cessation of breastfeeding consistent [12, 29, 30].

### Dynamics of the metaproteome during early childhood

**Fig. 2:**
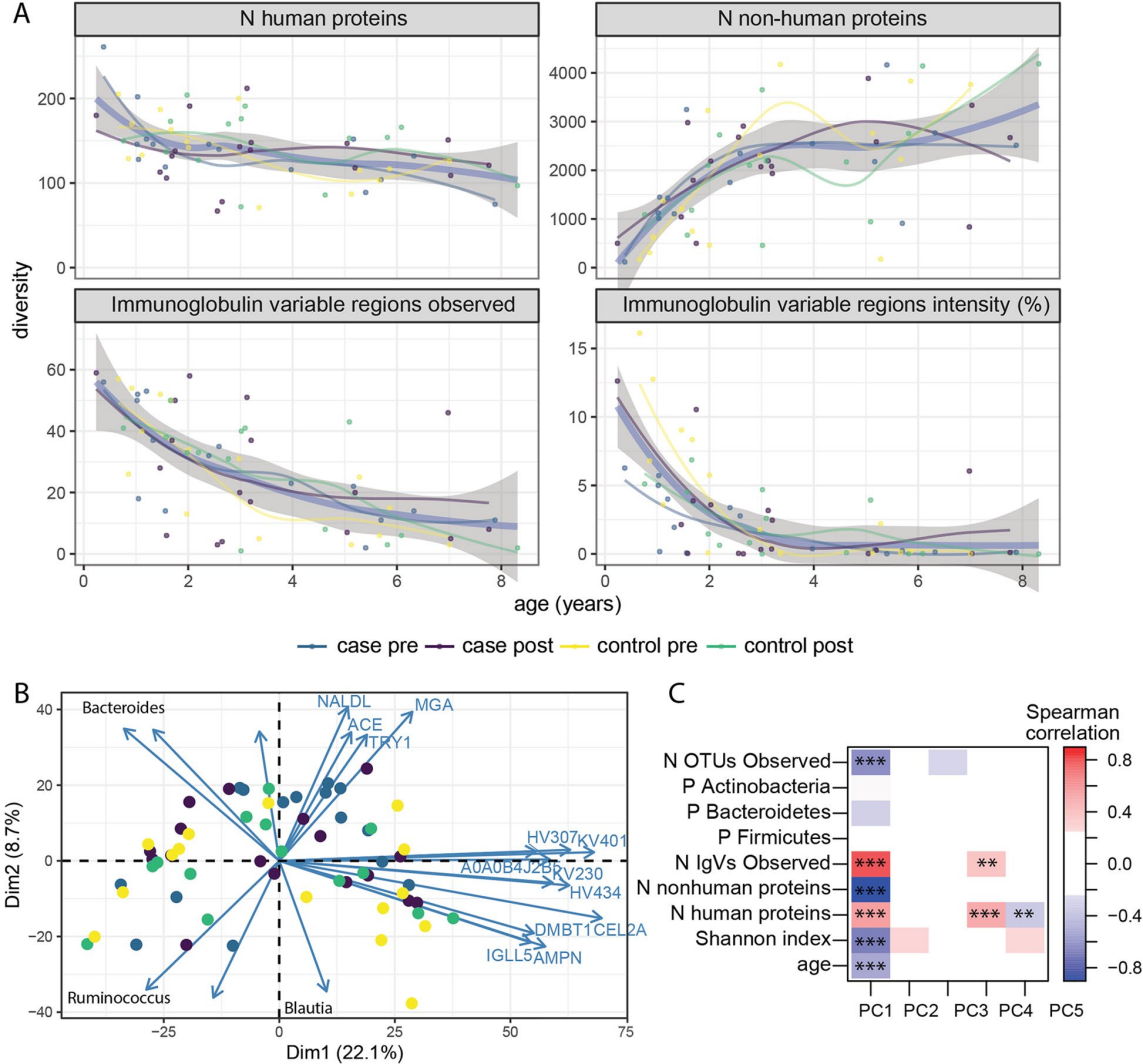
Dynamics of the metaproteome during early childhood. **A** The number of human proteins, non-human proteins, immunoglobulin-variable regions detected, and the proportion of immunoglobulin (IG)-variable regions among total protein intensity. For each panel, the thick blue curve represents all samples, the grey shaded are represents the 95% confidence interval, and the thinner curves represent each sample group. **B** PCA of the metaproteome shows PC1 and PC2 with contributing human proteins labeled individually. Genus of microbial proteins identified in each quadrant are indicated. **C** The correlation of the first 5 principal components with metrics from amplicon sequencing (phylum abundance, observed, Shannon) and metaproteomics (n human proteins, n non-human proteins). *p < 0.05, **p < 0.01, ***p < 0.001

Paralleling the increase in microbial diversity observed in amplicon sequencing, the total number of microbial proteins identified rose rapidly in the first three years of life, after which they stabilized (Fig. 2A). The number of microbial proteins was highly correlated to the number of OTUs detected by 16S sequencing (Supplementary Figure S1, *R* = 0.83, *p* < 0.001). The overall number of human proteins was stable across the range of ages within this study (Fig. 2A), although the number and combined intensity of immunoglobulin variable regions observed and immunoglobulin kappa and lambda light chain intensity decreased with age (Fig. 2A, Supplementary Figure S2). Interestingly, none of the class-specific immunoglobulin heavy chains significantly associated with age (Supplementary Figure S2), suggesting the individual variable regions may come from more than one antibody class. Neither the overall number nor intensity of human and microbial proteins were associated with case-control status (Fig. 2A, B and data not shown). Principal component (PC) analysis of the stool proteome revealed a strong association of age-related variables with the first principal component (Fig. 2B, C). PC1, which explained 22% of the variance, correlated with age, relative abundance of Bacteroidetes, the richness and evenness of microbial OTUs, and the number of human and microbial peptides identified (Fig. 2C). The number of human proteins and antibody-variable regions detected appear to play an important role in the data structure, as these are correlated with PCs 1, 4, and 5. None of the first 5 PCs were associated with case-control status.

Univariate analyses found 28 human and 45 microbial proteins were associated with the child’s age at sample collection (Supplementary Figure S3). The majority of human proteins, including 19 of the 27 immunoglobulins tested (*p* = 3 × 10^−10^), decreased with age, while the majority of microbial proteins increased. With the exception of the IG-κ and an IGλ-like constant chain, these immunoglobulin fragments were from antibody variable regions. Additional host proteins included MUC1, CLCA1, DMBT1 (also known as SALSA), and lactotransferrin (TRFL, also known as lactoferrin) which are involved in the maintenance of the gut barrier and anti-microbial defense [27, 28, 31, 32]. The microbial proteins associated with age include 9/36 (*p* < 0.001) of the glutamate dehydrogenases tested and these were predominantly derived from the Firmicutes phylum. The microbial gene content of proteins involved in glutamate metabolism has been observed to increase in a large study of children between 3 and 36 months old [33] and glutamate synthase was increased between ages 0 and 12 months in the TEDDY study [6]. Most of the other microbial proteins associated with age belong to ubiquitous pathways including glycolysis and were predominantly from Firmicutes phylum members.

### Gut microbiome and metaproteome associations with viral infections in early childhood

**Fig. 3:**
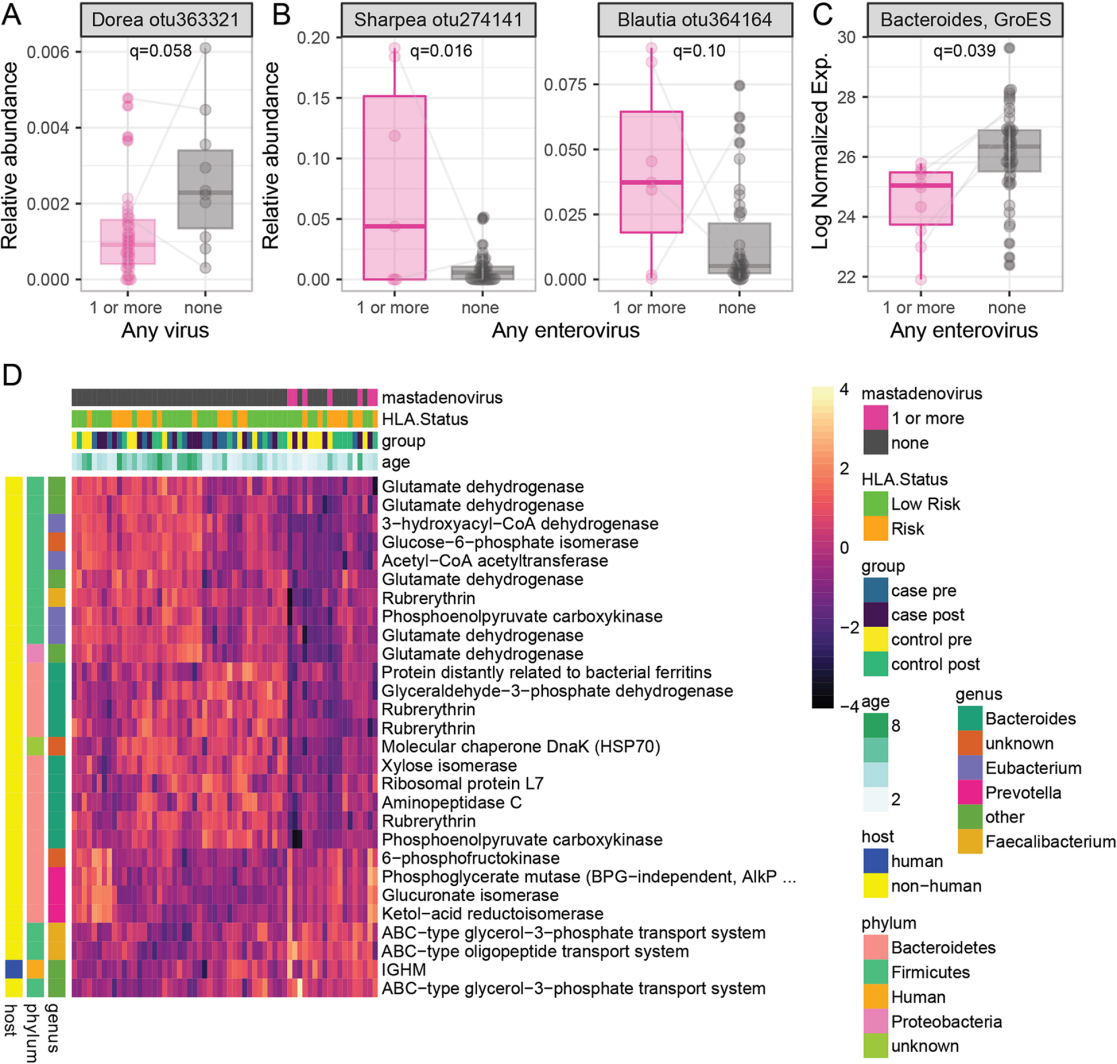
Microbial features associated with viral infection. Linear mixed models were used to identify OTU associated with **A** detection of any virus and **B** samples with any enterovirus. Linear mixed models were used to identify **C** proteins associated with the detection of any enterovirus and **D** proteins associated with any mastadenovirus. The 28 proteins associated with mastadenovirus infection are shown in a heatmap grouped using non-hierarchal clustering. Taxonomic and functional characteristics of the variables associated with each protein are indicated by the color in the legend

Next, we investigated the relationship between the gut virome, microbiome, and metaproteome using mixed-effect models. Separate models were used to evaluate the presence of any virus, any enterovirus or any mastadenovirus with microbial abundance. Exploratory analyses of norovirus and parechovirus were limited due to their sparsity. Positivity for enterovirus, but not other viruses, was associated with younger age (enterovirus positive samples 1.9 ± 1.1 years, enterovirus negative samples 3.6 ± 2.2 years, *p* < 0.001). Microbial diversity tended to be lower in virus positive samples, but these differences were not statistically significant when adjusted for age (*q* > 0.1, Supplementary Figure S4). A lower relative abundance of a *Dorea* OTU was associated with positivity to any vertebrate-infecting virus (*q* = 0.058, Fig. 3A). An OTU from *Blautia* and another from *Sharpea* were increased in enterovirus positive samples compared to those with no enterovirus (Fig. 3B). Individual microbial abundance was not associated with the presence of mastadenovirus.

Protein groups were then investigated for association with the presence of viruses. No proteins were associated with the detection of any virus (*N* = 50) in a sample compared to those with no virus detected (*N* = 11). A single bacterial protein, a Gro-Es heat shock protein from *Bacteroides*, was associated with enterovirus infection (Fig. 3C). This Gro-Es protein was not associated with age. In contrast, 28 microbial proteins were associated with the presence of mastadenovirus (Fig. 3D). These included 5 of the 36 tested glutamate dehydrogenases (GLUD1s, *p* = 0.02, *χ*^2^ test) and 4 out of 20 of the Rubrerythrins detected (*p* = 0.005), which were under-expressed in samples with presence of mastadenovirus. Three ABC-type transport proteins were also over-expressed in mastadenovirus positive samples. The GLUD1 proteins associated with mastadenovirus were distinct from those associated with age, changed in a consistent direction, and originated from a number of different Firmicutes and Proteobacteria. This suggests a functional change occurs in the microbiota rather than a change in the abundance of specific bacteria. Further supporting this idea, some *Faecalibacterium* derived proteins decreased (Rubrerythrin) while others (2 ABC-type transporters) increased in mastadenovirus-positive samples. Nearly half (9 of 20) of the proteins which were decreased in these samples came from *Bacteroides* while none of the 8 elevated proteins came from *Bacteroides*, suggesting some of the differences observed might be due to altered abundance and not function. Of the human proteins tested, only the heavy chain of IgM was elevated in samples with mastadenovirus. The large number of microbial proteins associated with mastadenovirus indicates that either this genus of viruses alters the functional state of the microbiome, perhaps through direct interaction or changes in the host immune system, or that the microbiome influences susceptibility to mastadenovirus infection.

To further explore functional changes in the metaproteome associated with viral infection, all non-host protein groups (*N* = 22,564) were aggregated by the Cluster of Orthologous Group (COG) of the lead protein. In general agreement with the results from individual protein groups, multiple COGs (*n* = 9) were associated with mastadenovirus infection (Supplementary Figure S5). This analysis confirmed the decrease of glutamate dehydrogenase observed in analysis of the individual protein groups with mastadenovirus as well as an increase in Glutamyl-tRNA synthetase. A number of microbial functions, including GLUD, were also associated with age (data not shown), but the association with mastadenovirus was independent of age. Together, these data suggest a functional remodeling of the gut microbiota accompanies mastadenovirus infection.

### Association of microbial abundance and metaproteome with islet autoimmunity

**Fig. 4:**
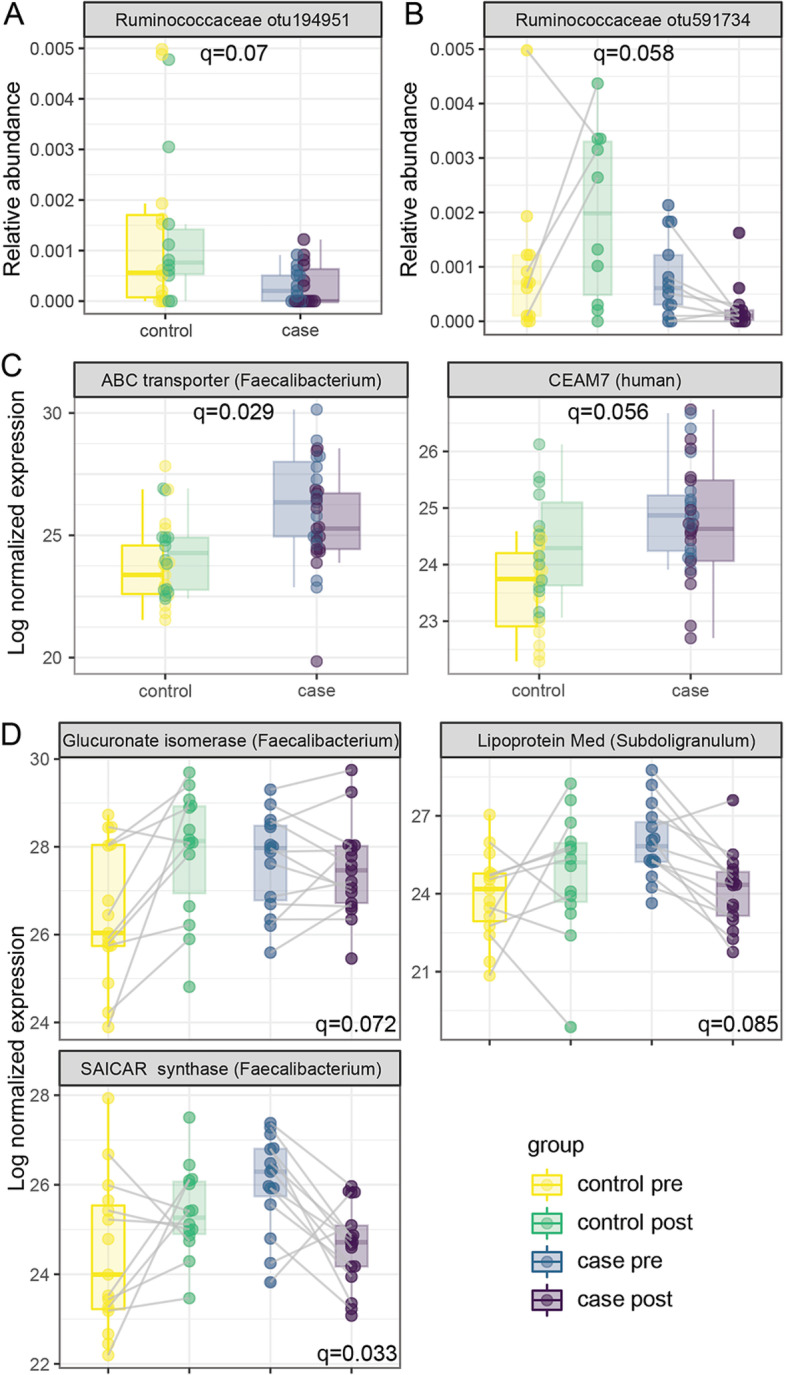
The microbiome and metaproteome are associated with islet autoimmunity. Linear mixed models identified **A** Ruminococceceae otu194951 associated with case/control status overall and **B** Ruminococceceae otu591734 associated with case/control status dependent on timepoint. **C** Proteins associated with case-control designation and **D** proteins associated with case/control status dependent on timepoint. Case pre: IAb+ subjects prior to seroconversion, case post: IAb+ subjects after seroconversion, control pre: IAb− subjects at first timepoint, control post: IAb− subjects at second timepoint

We investigated associations between islet autoimmunity and microbial or protein abundance. Univariate analysis identified a single unclassified Ruminococcaceae OTU was significantly more abundant in control children (Fig. 4A). An additional unclassified Ruminococcaceae OTU was identified in interaction tests to increase over time in controls but decrease post seroconversion in cases (Fig. 4B). Two proteins, human carcinoembryonic antigen cell adhesion molecule 7 (CEAM7, also known as CEA) and an ABC transporter from *Faecalibacterium Prausnitzii,* were detected at increased levels in cases compared to control children (Fig. 4C). CEAM7 (CEACAM7) is a cell adhesion molecule expressed on epithelial cells of the colon, rectum, and pancreatic duct [34, 35]. Three additional proteins were more abundant in case samples before seroconversion and decreased after seroconversion, while in control children they increased over time (case-time interaction *q* < 0.1, Fig. 4D). Two of these, a glucuronate isomerase and a SAICAR synthase, were from the *Faecalibacterium* genus within the Ruminococcaceae family. Glucuronate isomerase is involved in glucose and secondary metabolite degradation. SAICAR synthase is an enzyme involved in purine nucleotide biosynthesis. The third protein, lipoprotein Med, is an ABC-type transport protein and originated from the *Subdoligranulum* genus also within the Ruminococcaceae family. Together, these changes indicate an altered functional response within several members of the Ruminococcaceae family are associated with the onset of islet autoimmunity.

### Multi-omic factor analysis identifies latent factors associated with age, immunoglobulins, mastadenovirus, and islet autoimmunity

To explore broader relationships between the human proteome, the metaproteome, the virome, and microbial abundance, these datasets were integrated using multi-omic factor analysis (MOFA) [36]. Initially, a “view” indicating the presence or absence of viruses at the rank of genus was included in this integration. However, the virome explained very little of the variance (< 1%, Supplementary Figure S6) and was removed from subsequent models. Instead, the identified latent factors were tested for association with viruses in the same manner as in the univariate analyses. In the resulting model (referred to as model 1), six latent factors were selected with *R*^2^ > 0.05 (Supplementary Figure S7A, B). The first latent factor was strongly associated with age (*q* < 0.001) and other age-related variables including the number of OTUs observed (*q* < 0.001), the number of non-human proteins detected (*q* < 0.001), and the number of antibody variable regions (IGV) detected (*q* < 0.001) (Supplementary Figure S7B, C, Supplementary Table S3).

**Fig. 5:**
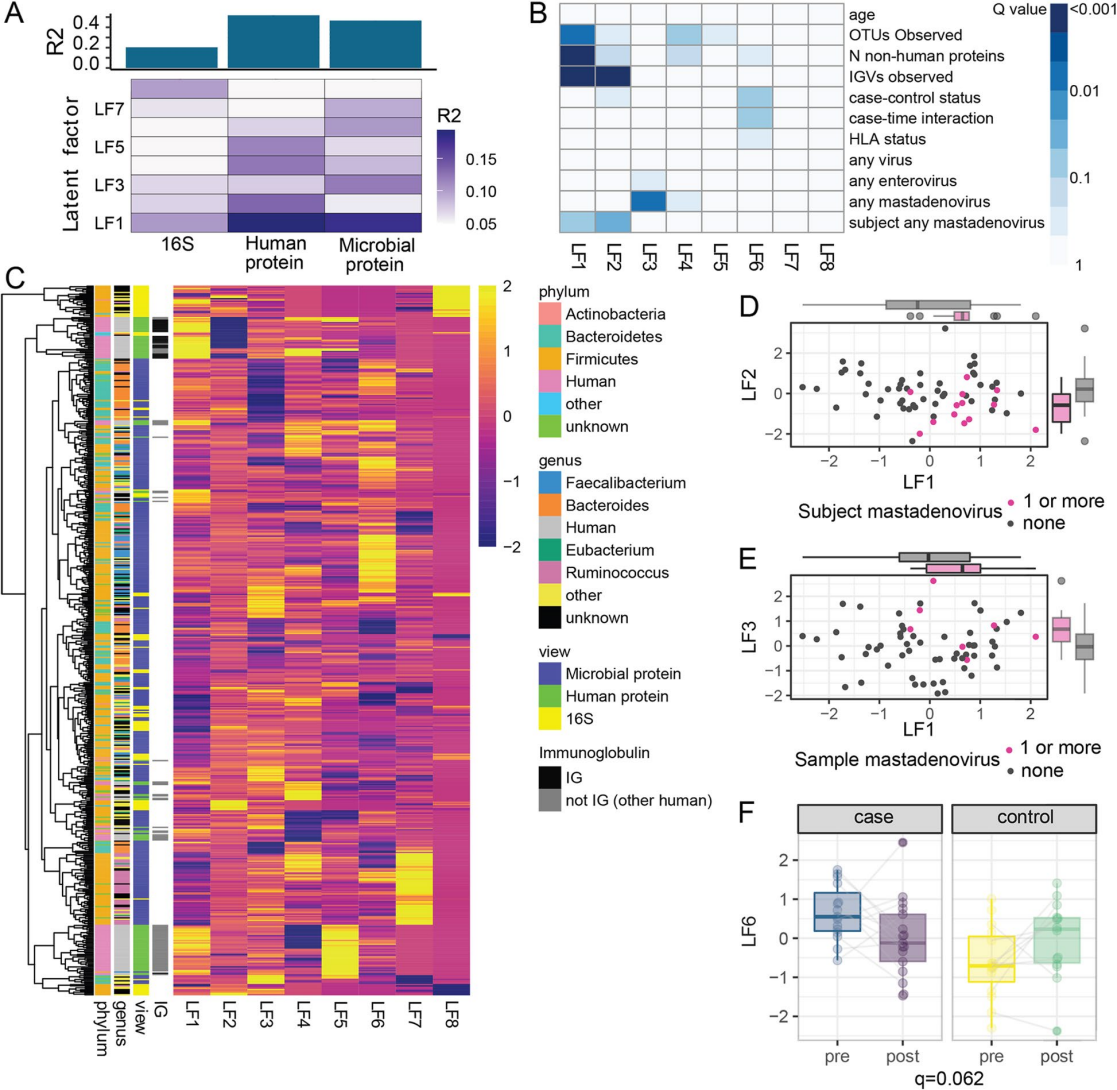
Multi-omic factor analysis model identifies latent factors associated with immunoglobulins, mastadenovirus and islet autoimmunity. This model was developed after cubic spline regression on age. **A** The total variance explained for each omic. **B** The association of the latent factors (LFs) with other variables with q-values from a linear mixed model adjusted for age. **C** Hierarchical clustering of the weights for each LF for those with an absolute weight > 1 for any factor. Taxonomic and functional characteristics of the variables in **C** are indicated by the color in the legend, and clusters of variables with common features are apparent in the annotated dendrogram. **D** Scatterplots of LF1 versus LF2 and **E** LF1 versus LF3 according to the presence of mastadenovirus in subjects or samples. **D** LF6 according to case-control designation and timepoint. Cases vs control *q* = 0.09, case-time interaction *q* = 0.062

**Fig. 6:**
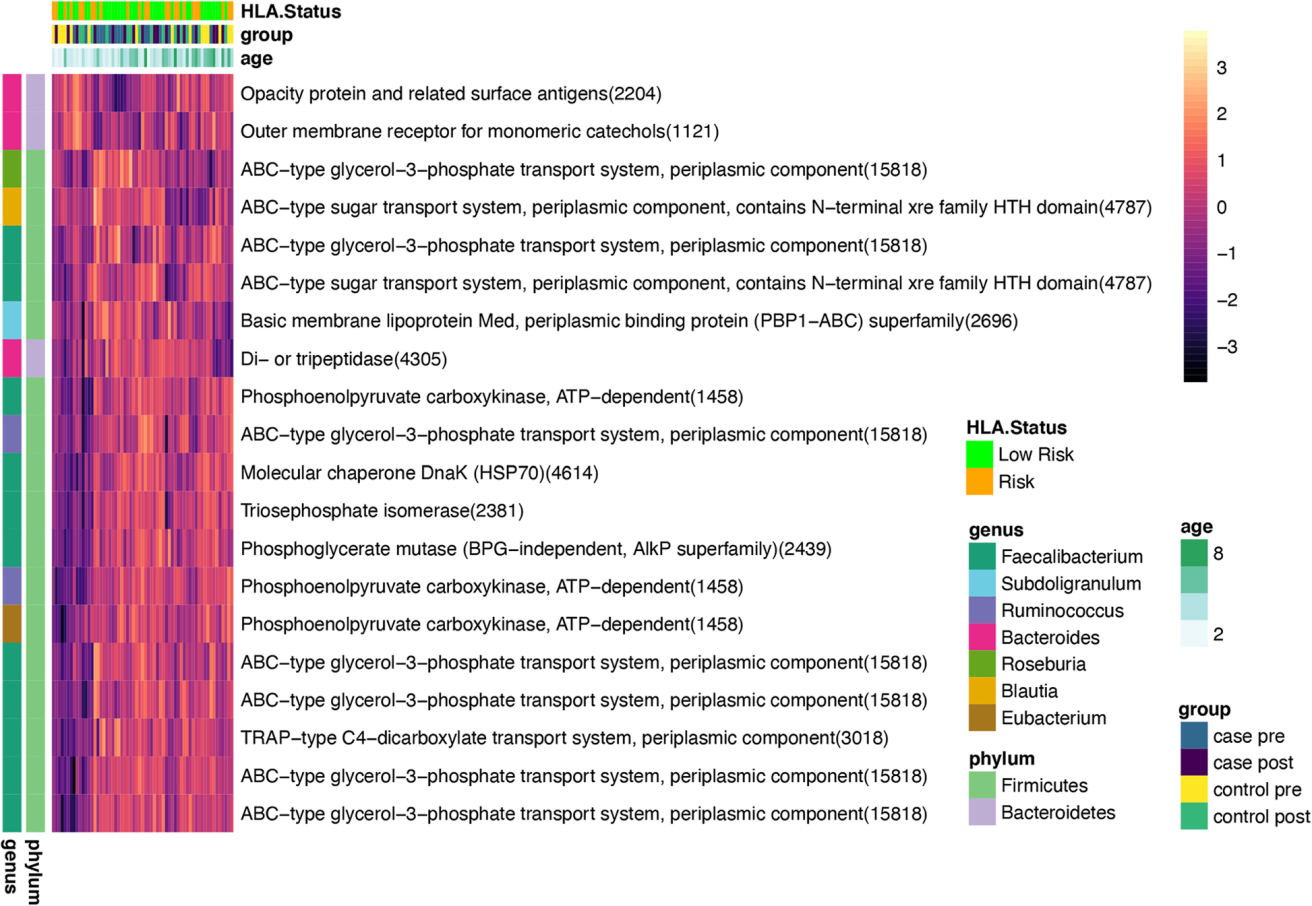
Top 20 variables associated with LF6 from multi-omic factor analysis model associated with case-control status. Hierarchical clustering of normalized intensities of the 20 variables with the strongest influence on LF6. These were all microbial proteins and included 11 derived from *Faecalibacterium*, and 9 ABC-type transport proteins. Taxonomic and functional characteristics of the proteins are indicated by the color in the legend. ABC-type glycerol-3-phosphate transport system, periplasmic component (15818) includes this protein from five different members of the genus *Faecalibacterium.*

To account for the non-linear effect of age on the microbiome, human proteome, and microbial proteome, a second MOFA analysis was performed, first removing the variance due to age using the residuals from a cubic spline regression for each variable as previously reported in single-omic studies [5, 15]. This model (referred to as model 2), identified 8 latent factors with *R*^2^ > 0.05, (Fig. 5A, B). As expected, LF1 was no longer associated with age (Fig. 5B, *R*^2^ = 0.00), but it was still associated with the number of OTUs observed, the number of non-human proteins detected, and the number of IGV detected. A strong effect of individual IGV fragments on LF1 and LF2 was still apparent (Fig. 5B, C, Supplementary Table S4), suggesting antibodies play an important role in shaping the microbiome and metaproteome. LF1 and LF2 were both associated with subjects that had a mastadenovirus infection at either timepoint while LF3 was associated with samples that had a mastadenovirus infection (Fig. 5B, D, E). No latent factors were associated with birth delivery mode (vaginal versus Caesarian delivery). The distribution of LF6 differed between cases and controls (*q* = 0.09) and displayed significant case-time interaction (*q* = 0.06, Fig. 5F). The microbial abundance estimates from amplicon sequencing explained a negligible amount of the LF6 variance (*R*^2^ = 7 × 10^−5^). Closer inspection of the top 20 proteins in LF6 revealed strong representation of proteins from the genus *Faecalibacterium* (11 proteins) and *Bacteroides* (3 proteins) (Fig. 6, Supplementary Table S5). Interestingly, while *Faecalibacterium* proteins tend to decrease post-seroconversion in cases and increase in controls over time, *Bacteroides* showed the opposite trend. Strikingly, there were 9 ABC-type transport proteins within the top 20 proteins (Fig. 6, Supplementary Table S5), suggesting alterations in transport of glycerol-3-phosphate or sugars in children that develop islet autoimmunity, possibly indicating altered substrate utilization by the bacteria. In summary, the global expression profiles of microbial and human proteins identified, in an unsupervised manner, latent factors which differ between children who develop islet autoimmunity and those who do not. The fact that microbial proteins rather than microbial taxa, human proteins, or viruses associated with the onset of islet autoimmunity suggests that the functional activity of the microbiota may be a key factor associated with seroconversion.

## Discussion

Although both viruses and the gut microbiome are believed to play a role in the pathogenesis of T1D and both have been investigated in large cohorts such as the TEDDY study [6, 19, 37], integrated multi-omic analyses of the two has not previously been reported. Here, we examined relationships between the metaproteome, microbiome, and virome in the stool of children collected before and at the onset of islet autoimmunity. We have made the following observations: (i) while the increasing diversity of the gut microbiome in early childhood strongly correlated with increasing diversity within the metaproteome, there was a marked decline in the number and abundance of host antibodies in stool over the same time period; (ii) intestinal infection with mastadenovirus but not with enterovirus was accompanied by a profound remodeling of the gut microbiome functional response; (iii) the onset of islet autoimmunity was accompanied by a decline in the abundance of members of the Ruminococcaceae family as well as several proteins they produce; (iv) unsupervised latent factor integration methods revealed a network of proteins produced by the genus *Faecalibacterium*, with particular enrichment for ABC-type transport proteins that were initially elevated but decreased over time in the children that developed islet autoimmunity.

Multiple studies have shown previously that the overall diversity of the gut microbiota rapidly increases over the first years of life, stabilizing by 2–3 years of age to an ‘adult-like’ consortia [24, 29, 30, 33]. For the first time, we show that this increase in overall microbial diversity is paralleled by an increase in the number of distinct microbial proteins detected and this is highly correlated with the number of OTUs observed. Strikingly, however, the number and total abundance of host antibody fragments rapidly decreased across the same developmental period while human proteins overall did not display this pattern. One possible explanation for this decline is that the initial exposure to new microbe and food antigens during infancy results in B cell activation and antibody secretion into stool. As the immune system matures and is tolerised to these commensal bacteria and harmless food antigens, antibody production in the gut may then be reduced. This idea is supported by evidence from others showing that the frequency of B cells expressing gut-homing receptors α4β7 is highest at 1–4 months of age and then declines with age [38]. In another study, the number of B cells per mL of peripheral blood peaked at 4 months of age and then declined with increasing age, while CD27^+^ memory B cells peaked at 18 months of age and then declined by adulthood [39]. Colonization of infants with *Escherichia coli* and/or *Bifidobacteria* correlated with higher numbers of CD27^+^ memory B cells during infancy compared to those without these taxa, suggesting that colonization with these members of gut microbiota was influencing the systemic B cell response [39]. Our data support that there is an early, profound intestinal antibody response to the introduction of new antigens in the gut during early infancy.

In the TEDDY study, analysis of the gut virome of infants at risk of developing T1D found evidence of a protective effect of infection with human mastadenovirus C and risk of future development of islet autoimmunity [19]. In addition, a large body of molecular and epidemiological data support the role of enteroviruses as key environmental triggers of islet autoimmunity and risk factor for T1D [4, 16, 19, 23]. Here, we show that mastadenovirus infection had a strong overall correlation with the structure of the stool metaproteome. While mastadenovirus is among the most common viruses detected in the gut of children [19], infections are typically asymptomatic or cause only mild respiratory symptoms, accounting for 5% of symptomatic upper respiratory infections and 14% of symptomatic lower respiratory infections [40, 41]. In the gut, human adenoviruses (which are all mastadenoviruses) can cause persistent infection leading to shedding in stool [42] and can cause gastroenteritis [43], with type C adenoviruses most commonly associated with persistent infection [44]. To date, no studies have reported an association between mastadenovirus and the gut microbiome in humans. Recently it was shown that naturally occurring adenovirus infection in non-human primates was associated with a profound shift in the microbial community with 87 OTU significantly altered and an overall increase in the phyla Firmicutes and class Clostridia observed in infected animals [45]. The mechanism behind a protective link between mastadenovirus C infection and the risk of islet autoimmunity is unknown with various mechanisms postulated including competitive interactions between different viruses [19]. Our data raise the intriguing possibility of an interaction between the function of the gut microbiota and mastadenovirus infection playing a role in T1D risk.

Associations between the gut microbiota and the onset of islet autoimmunity have been described in multiple studies, with substantial variability in the specific taxa associated with disease risk in each cohort [6, 12, 13, 15, 25, 26, 29]. This variation in species composition may be due to geographic differences between the cohorts. Another explanation is that common functional pathways are altered rather than specific taxa, which are highly variable. Here, we found altered abundance of 2 OTU from the Ruminococcaceae family that were lower in children with islet autoimmunity. More profoundly, we identified a cluster of proteins associated with LF6 from *Faecalibacterium* and proteins involved in carbohydrate transport and metabolism that were initially elevated but then tended to decrease after the onset of islet autoimmunity in contrast to the controls where these proteins increased over time. Previously, we used metaproteomics to investigate functional changes which may underlie dysbiosis associated with islet autoimmunity or T1D in a cohort from Colorado [46]. In that cohort, we similarly identified a cluster of nine proteins derived from *Faecalibacterium prausnitzii* that were associated with features that distinguished healthy controls and first-degree relatives without autoimmunity in comparison with individuals with islet autoimmunity [46]. Of these *F. prausnitzii* proteins, three were involved in membrane-transport systems for sugars including the glycerol-3-phosphate transport system, similar to the *Faecalibacterium* derived proteins that contributed to the model we describe here. This suggests that similar relationships may exist between altered function of *Faecalibacterium* genus members and the onset of islet autoimmunity in this cohort.

The link with carbohydrate transport systems suggests there may be an alteration in carbon utilization by *Faecalibacterium* in children with islet autoimmunity. The ABC transporters identified were linked to sugar and glycerol-3-phosphate transport. Glycerol-3-phosphate can be derived from glycolysis or lipid metabolism [47, 48]. Functional potential indicative of such behavior was observed by Kostic and colleagues using a densely sampled case-control cohort of children under 3 years of age [25] and a second study which predicted increased ABC transport using PICRUST in children that later developed T1D [49]. In the Kostic study, the metagenome of children who developed T1D contained more genes for the multiple-sugar transport system and fewer genes for amino acid biosynthesis than the metagenomes of healthy children, leading the authors to speculate a functional shift had occurred from nutrient synthesis to nutrient uptake [25]. Of interest, mastadenovirus infection was also associated with elevation of three similar (but not identical) ABC-type transporters, two from *Faecalibacterium* and one from an unknown taxon. This suggests that mastadenovirus may influence similar functional pathways to those associated with islet autoimmunity. Further studies are needed in larger and more densely sample cohorts to better understand these relationships.

Many other studies have implicated *Faecalibacterium* members, particularly *F. prausnitzii* as having a fundamental role in human health [50–52]. *F. prausnitzii* is one of the most abundant producers of the beneficial short-chain fatty acid (SCFA) butyrate in the healthy human gut [53], in addition to producing other anti-inflammatory secretory components [50]. A reduction in bacteria capable of fermenting dietary fiber to produce SCFAs has been identified as one of the most consistent features of dysbiosis associated with T1D [6, 11, 12, 15]. *Faecalibacterium* and unclassified *Ruminococcaceae* OTUs were observed at lower relative abundance in children who develop autoimmunity in some small case control studies [49, 54] and in the much larger TEDDY longitudinal study [29]. After the onset of clinical T1D, *Faecalibacterium* abundance was negatively correlated with HbA1c [49, 55] and serum levels of zonulin, a common biomarker for gut barrier function [49]. Further investigation is required to explore the association of specific *Faecalibacterium* functional changes with autoimmunity.

The main limitations of our study were the small sample size and availability of only two timepoints per participant, some without a pre-seroconversion timepoint. This meant that the number of samples with a detected infection with any given virus was low. We may also have missed viral infections that were cleared rapidly but still had a lasting impact on the microbiota or the host. This limited our ability to investigate microbiome associations with many individual viruses such as norovirus or the various subtypes of mastadenovirus. We also did not investigate other parameters that may drive changes in the gut microbiota such as diet or antibiotic use due to the complexity of these types of data and small sample size. A limitation of shotgun proteomics is a low overall depth of coverage. Only a fraction of the bacterial proteins predicted to be present are abundant enough for detection. Furthermore, we analyzed the ‘soluble’ fraction of the stool as this is enriched for human proteins, but also bias’s the detection of microbial proteins toward those from gram-negative bacteria [56]. A strength of our study was the use of unsupervised data integration methods to uncover the relationships in the data with islet autoimmunity and viral infection. Providing greater confidence in our novel approach, this method validated some of the relationships we had previously identified in another cohort using a supervised approach [46]. We also present the first microbiome and metaproteome data from the southern hemisphere in children with islet autoimmunity.

## Conclusions

In conclusion, we demonstrate the interrelationship between the gut microbiota, metaproteome and virome in young children. We have uncovered a previously unrecognized association of mastadenovirus infection with the function of the gut microbiota, which may play a role in disease risk. Strikingly, we found further evidence of a switch in *Faecalibacterium* function may be associated with the onset of islet autoimmunity. This lends evidence that an altered gut microbiota is involved in the pathogenesis of T1D and therapeutic strategies aimed at re-training the gut microbiota may be a promising approach for a therapy for preventing T1D. Further studies in larger cohorts such as the TEDDY study or the Australian ENDIA study may further elucidate these findings.

## Methods

### Participant characteristics

Participants were from the Australian Viruses in the Genetically at Risk (VIGR) prospective birth cohort, a longitudinal observational study of children who had a first-degree relative with T1D [20, 23]. Feces were examined in twenty subjects positive for 1 or more islet cell autoantibodies to GAD65, insulin, or IA2 in 2 consecutive visits (case subjects). A fecal sample was available for analysis following the detection of seroconversion and in the preceding ~ 1 year for 12 subjects; fecal samples were available only at the time of seroconversion for 5 subjects and only preceding seroconversion in 3 subjects (case subjects). For each case subject, an age-, gender-, and HLA-matched subject was selected and the corresponding timepoints evaluated (control subjects). One case had an unknown HLA risk status, this was imputed to have low risk for models and graphs involving HLA status. Virome sequencing using VirCapSeq-VERT for all 64 specimens has been previously reported [20]. Three controls did not have a specimen available corresponding to the post-seroconversion timepoint for metaproteomics or 16S amplicon sequencing. Sequencing was unsuccessful in twelve additional pre-seroconversion case specimens (described below), yielding a total of 49 samples available for analysis of the microbiome and 61 samples for metaproteomics. Fifty percent of subjects were male; 40% had a high-risk HLA haplotype as previously defined [20]; islet auto-antibodies were detected at an average age of 3.4 (± 2.2) years; and on average 1.4 (± 0.6) years passed between the timepoints investigated (Supplementary Table S1). Samples were collected between 2006 and 2015 and stored at − 80 °C.

### Virome sequencing

Methods for virome sequencing and the association of specific viruses with islet autoantibodies in this cohort have been reported [20] and used a capture approach where viral sequences were captured by over 2 million probes designed to isolate DNA from viral taxa known to infect vertebrates (VirCapSeq-VERT) [57]. For the current analysis, viruses with greater than 100 reads were considered detected. To investigate the association of the virome with the metaproteome and microbial abundance, the following categories were considered for each sample: (1) detection of any virus, (2) detection of any enterovirus, and (3) detection of any mastadenovirus. The same criteria were applied for each *subject,* giving a total of 8 possible classifications.

### Protein extraction, digestion, and purification

The slurry remaining after preparation of viral DNA was thawed on ice and resuspended by gentle vortexing for 30 s in an equal volume of ice-cold phosphate buffered saline (PBS). Each sample was then split into two new tubes using a wide-bore 1 mL tip to ensure solids were evenly distributed. Peptides were then prepared according to the human enrichment protocol as described [56] with minor modifications, most notably the exclusion of PNGase F digestion. The aliquot for proteomics was centrifuged at 8000×*g* for 10 min at 4 °C to pellet debris and intact bacterial cells. The supernatant was transferred to a new tube and centrifuged under the same conditions. Total protein (20 μg), as estimated by the direct detect spectrometer, were then solubilized, reduced, and alkylated at 95 °C for 5 min in a solution containing 1% sodium deoxycholate, 10 mM (tris[2-carboxyethyl]phosphine) and 40 mM 2-Chloroacetaldehyde in 100 mM Tris pH 8. This solution was diluted 1:10 in water and proteins were digested overnight at 37 °C by addition of modified pig trypsin (Promega Madison, WI, USA) at a protein:trypsin ratio of 50:1 (w/w). Detergents were precipitated by addition of 10% trifluoroacetic acid, the supernatant was transferred to a fresh plate, and salts were removed using C18 tips (Glygen, Columbia, MD, USA) on a Bravo liquid handler. The eluent was evaporated with centrifugation under vacuum and tryptic peptides were resuspended in 0.1 % formic acid for mass spectrometry. Assays were performed blinded to clinical information and results of virome sequencing.

### Mass spectrometry and data processing

Tryptic peptides were used for liquid chromatography–tandem mass spectrometry analysis on a Q Exactive mass spectrometer (Thermo Fisher Scientific, Waltham, MA, USA). Peptides were separated on an EASY-Spray analytical column (Thermo Fisher Scientific) using a 90-min gradient from 3 to 25% acetonitrile. MS1 was acquired for ions with a mass/charge ratio of 350–1400 and the top 20 ion were subjected to MS2. Each sample was analyzed in duplicate. Peptide spectrum matching, protein inference, grouping, and quantification were performed using the MetaPro-IQ [58] strategy implemented in MetaLab version 1.1 [59] and MaxQuant version 1.6.3.4. The integrated reference catalog of the gut microbiome from Li et al [60] was used for the initial search. Human proteins were retrieved from Uniprot. Spectral clustering was disabled during database generation. Carbamidomethylation of cysteine and oxidation of methionine were included as fixed and variable modifications, respectively, during MaxQuant searches. Spectra from replicate runs were merged during the MaxQuant pipeline. Based on previous reports, proteins were filtered to include those detected in at least 50% of samples [61], intensities were normalized by variance stabilization [62], and missing values were imputed with BPCA [63]. All proteins were included to determine the summed expression of Clusters of Orthologous Groups (COGs).

### 16S Amplicon sequencing and data processing

Bacterial abundance in stool was determined by amplicon sequencing of the V6–V8 variable region (nucleotides 926-1392) of the 16S rRNA gene [64]. Lysis buffer containing 50 mM Tris-HCl, 500mM NaCl, 50mM EDTA, and 4% sodium dodecyl sulfate was added to each aliquot of fecal sample. A 50:50 mixture of 1 mm and 0.1 mm silica beads (total 0.4 g, Daintree Scientific) was then added to each tube, and bacterial cells were lysed on a tissue homogenizer using three rounds of shaking at 5000 Hz for 45 s. DNA was prepared from the lysate using the automated Maxwell 16 Research System (Promega) with the Blood LEV kit (Promega) following the manufacturer’s instructions. Resulting nucleic acids were incubated at 37 °C for 30 min with 20 μg of RNAse A to remove RNA. The V6–V8 region of the 16S gene was then amplified using primers AAACTYAAAKGAATTGACGG and ACGGGCGGTGTGRC with Illumina specific adapters (Integrated DNA Technologies, NSW, Australia) and Q5 High-Fidelity DNA Polymerase (New England BioLabs, Inc.; Ipswich, MA, USA). Thirty-three cycles of PCR were performed using the following conditions: melt at 95°°C for 30 s, anneal at 65 °C for 40 s, and extend at 72 °C for 40 s. Amplicons were purified using AMPure XP beads and Nextera XT indices (Illumina) were added by 10 rounds of PCR. Indexed amplicon libraries were then purified using AMPure XP beads (Beckman Coulter, Inc.; Brea, CA, USA), pooled to an equimolar concentration of 10 nM, and sent to the Australian Center for Ecogenomics (ACE, Brisbane, Australia) for sequencing on an Illumina MiSeq. Assays were performed blinded to clinical information and results of virome sequencing.

Operational taxonomic units (OTUs) were identified using the UPARSE analysis pipeline for OTU calling and taxonomy prediction with percent identity of 0.97 and a minimum cluster size of 2. The GreenGenes 16S RNA Gene Database version 13_8 was used for taxonomy prediction. Reads were filtered for quality control using a MAXEE score of 1. Filtered reads were used to generate the OTUs. Reads not passing quality control were truncated to a max length of 200 bases and used in the step assigning reads to OTUs. Ten samples showed abnormally high levels of a Methanobrevibacter OTU, likely due to contamination of shared lab equipment. Multiple previous attempts to amplify these samples were unsuccessful. These ten samples and two additional samples with failed sequencing reactions (< 100 reads) were removed from downstream analyses. Methanobrevibacter abundance remained slightly higher than anticipated after this adjustment (0.5 ± 1.5%, < 0.1% in iHMP study [65]) and was excluded from further analysis.

Due to the small number of samples, the number of OTUs was reduced using an amalgamation approach based on taxonomic hierarchy for dimension reduction. The amalgamation method used is a simplified version of that proposed by others [66, 67]. The number of OTUs was reduced by combining those accounting for less than 0.1% of the reads in at most 15% of samples to their common parent taxa. Resulting OTUs accounting for more than 0.1% of reads in at least 15% of samples were left unaltered while those that did not meet these criteria were combined to their common parent taxa. This process of “collapsing” was repeated three times, and OTUs which still failed to meet these criteria were excluded, reducing the number of OTUs from 510 to 120. This strategy is intended to reduce the number of variables while minimizing the loss of potentially meaningful data.

### Statistical analyses

The microbiome and metaproteome were first evaluated separately to identify proteins or bacteria associated with the presence of autoantibodies (case vs control) or viruses (any vs none; any enterovirus vs no enterovirus; any mastadenovirus vs no mastadenovirus). After filtering and collapsing as described above, each OTU (*n* = 120) or protein (*n* = 730) was included in a linear mixed model. The metaproteomes of 61 specimens from 40 subjects were analyzed. The 85,000 unique peptides detected were combined into 22,564 microbial protein groups and 459 human protein groups. Participant ID was included as a random effect. Age at collection (years), HLA risk (high or low), and islet autoantibody outcome (case/control) were included as fixed effects. An indicator for viral detection was included in separate models for each of the categories described above. False discovery rate (FDR) was used to correct for multiple hypotheses, and a *q* value of 0.1 was considered statistically significant. The arcsin of the square root of total-sum-scaled data was used for modeling of microbial abundances. Principal coordinate analysis of the Bray Curtis distances and principal components were used to visualize the microbial and metaproteomic data, respectively.

Multi-omic factor analysis (MOFA) was applied to integrate the datasets [36]. Thi**s** method has several attributes which make it suited to this dataset. Firstly, MOFA allows inclusion of samples which are missing one of the data types. Secondly, it can model binary data types, such as the presence or absence of viruses, following a Bernoulli distribution. Thirdly, MOFA is an unsupervised technique which can identify shared dimensions across multiple omics. These linear combinations of variables, known as latent factors, are analogous to principal components and allow a means of dimension reduction. To evaluate their association with autoantibodies and other phenotypes, latent factors explaining more than 5% of the variance were included in mixed models as described for the individual proteins and taxa. To account for the non-linear effect of age, a second MOFA model was developed using the residuals from a cubic spline regression and the resulting latent factors were evaluated in the same manner.

## Supplementary Information

**Additional file 1: Supplementary Figure S1.** The number of OTUs observed is correlated to the number of nonhuman proteins detected. Each color represents a subject, and the lines indicate repeated measures for representative individuals. Correlation (*R*=0.83) was determined for repeated measures (*p*<0.001). **Supplementary Figure S2.** Immunoglobulin heavy and light chain constant regions detected in stool related to age. For each panel, the thick blue curve represents all samples, the grey shaded are represents the 95% confidence interval, and the thinner curves represent each sample group. Case pre: IAb+ subjects prior to seroconversion, case post: IAb+ subjects after seroconversion, control pre: IAb- subjects at first timepoint, control post: IAb- subjects at second timepoint. IGLL5 and IGKC were significantly associated with age (q<0.1). **Supplementary Figure S3.** Stool proteins associated with age. Linear mixed models identified 45 microbial and 28 human proteins associated with age. Taxonomic and functional characteristics of the proteins are indicated by the color in the legend represented as a heatmap grouped using non-hierarchal clustering. **Supplementary Figure S4.** Microbial diversity is not altered in the presence of virus. The Observed number of OTUs and the Shannon index tend to be lower in samples which have an infection, but these differences are not significant when adjusted for age (all q>0.1). **Supplementary Figure S5.** Microbial functions associated with mastadenovirus. Proteins were aggregated by their Cluster of Orthologous Group (COG) assignments and evaluated for association with viral infection. Summed protein intensity from nine COGs shown are associated with the presence of mastadenovirus (q<0.1). **Supplementary Figure S6.** The virome explains little of the total variance following data integration. MultiOmic Factor Analysis (MOFA) was used to integrate the virome, human proteome, microbial proteome, and 16S abundance estimates. (A) The total variance explained by each omic or “view”, and (B) the variance explained for each latent factor. **Supplementary Figure S7.** MultiOmic Factor Analysis model 1 identifies latent factors associated with age, mastadenovirus and islet autoimmunity. (A) The total variance explained for each view and contribution to each latent factor (LF), along with a representation of the weights for latent factor for those with an absolute weight >1 to any factor. (B) The association of the latent factors with other variables. Q-values from a linear mixed model adjusted for age.

**Additional file 2: Supplementary Table S1.** Cohort characteristics. **Supplementary Table S2.** OTU associated with age. **Supplementary Table S3.** MOFA loadings contributing to latent factors 1-6 for model 1. **Supplementary Table S4.** MOFA loadings contributing to latent factors 1-8 for model 2. **Supplementary Table S5.** Top 20 loadings for latent factor 6 from model 2.

## Data Availability

The 16S rRNA amplicon sequencing data has been deposited in the Sequence Read Archive (SRA) database, BioProject accession number PRJNA822783. The mass spectrometry proteomics data have been deposited to the ProteomeXchange Consortium via the PRIDE partner repository with the dataset identifier PXD032997.
