# Supplementary material for: Multi-omic interactions in the gut of children at the onset of islet autoimmunity
Source: Microbiome. 2022 Dec 16;10:230. doi: 10.1186/s40168-022-01425-6 (PMC9756488; doi:10.1186/s40168-022-01425-6)
Supplement: Supplementary file 2 — Additional file 1: Supplementary Figure S1. The number of OTUs observed is correlated to the number of nonhuman proteins detected. Each color represents a subject, and the lines indicate repeated measures for representative individuals. Correlation (R=0.83) was determined for repeated measures (p<0.001). Supplementary Figure S2. Immunoglobulin heavy and light chain constant regions detected in stool related to age. For each panel, the thick blue curve represents all samples, the grey shaded are represents the 95% confidence interval, and the thinner curves represent each sample group. Case pre: IAb+ subjects prior to seroconversion, case post: IAb+ subjects after seroconversion, control pre: IAb- subjects at first timepoint, control post: IAb- subjects at second timepoint. IGLL5 and IGKC were significantly associated with age (q<0.1). Supplementary Figure S3. Stool proteins associated with age. Linear mixed models identified 45 microbial and 28 human proteins associated with age. Taxonomic and functional characteristics of the proteins are indicated by the color in the legend represented as a heatmap grouped using non-hierarchal clustering. Supplementary Figure S4. Microbial diversity is not altered in the presence of virus. The Observed number of OTUs and the Shannon index tend to be lower in samples which have an infection, but these differences are not significant when adjusted for age (all q>0.1). Supplementary Figure S5. Microbial functions associated with mastadenovirus. Proteins were aggregated by their Cluster of Orthologous Group (COG) assignments and evaluated for association with viral infection. Summed protein intensity from nine COGs shown are associated with the presence of mastadenovirus (q<0.1). Supplementary Figure S6. The virome explains little of the total variance following data integration. MultiOmic Factor Analysis (MOFA) was used to integrate the virome, human proteome, microbial proteome, and 16S abundance estimates. (A) The total variance ex [file 40168_2022_1425_MOESM1_ESM.pdf]

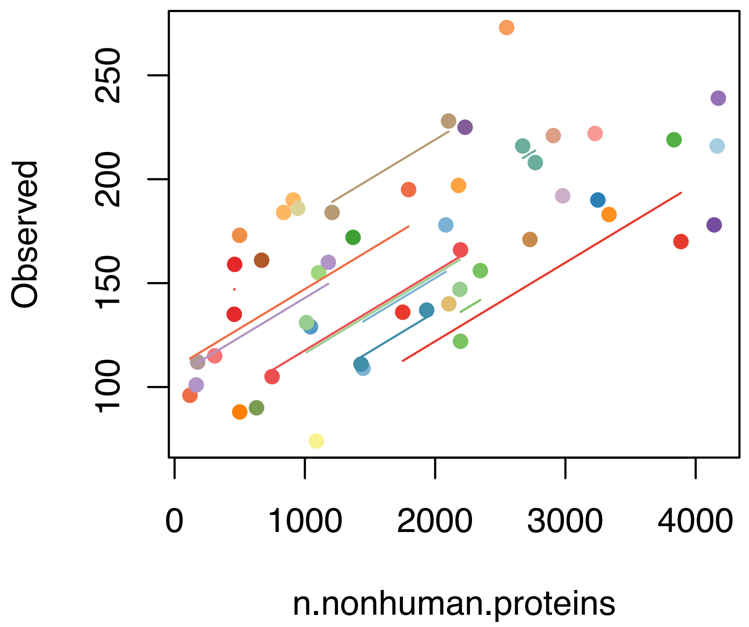

Supplementary Figure 1: The number of OTUs observed is correlated to the number of nonhuman proteins detected. Each color represents a subject, and the lines indicate repeated measures for representative individuals. Correlation (R=0.83) was determined for repeated measures.

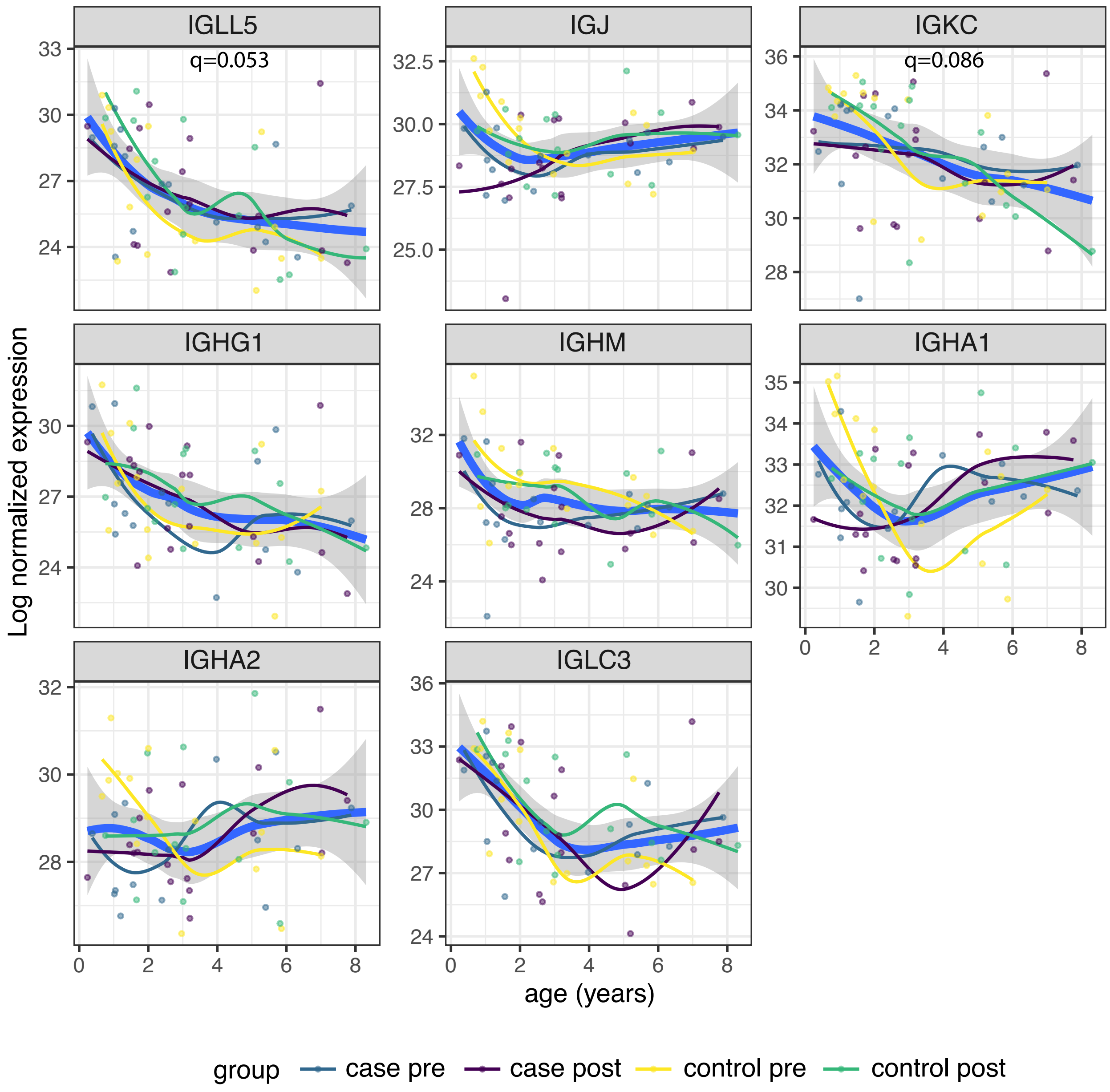

Supplementary Figure 2: Immunoglobulin heavy and light chain family proteins related to age. For each panel, the thick blue curve represents all samples, the grey shaded are represents the 95% confidence interval, and the thinner curves represent each sample group. Case pre: IAb+ subjects prior to seroconversion, case post: IAb+ subjects after seroconversion, control pre: IAb- subjects at first timepoint, control post: IAb- subjects at second timepoint. IGLL5 and IGKC were significantly associated with age (q<0.1).

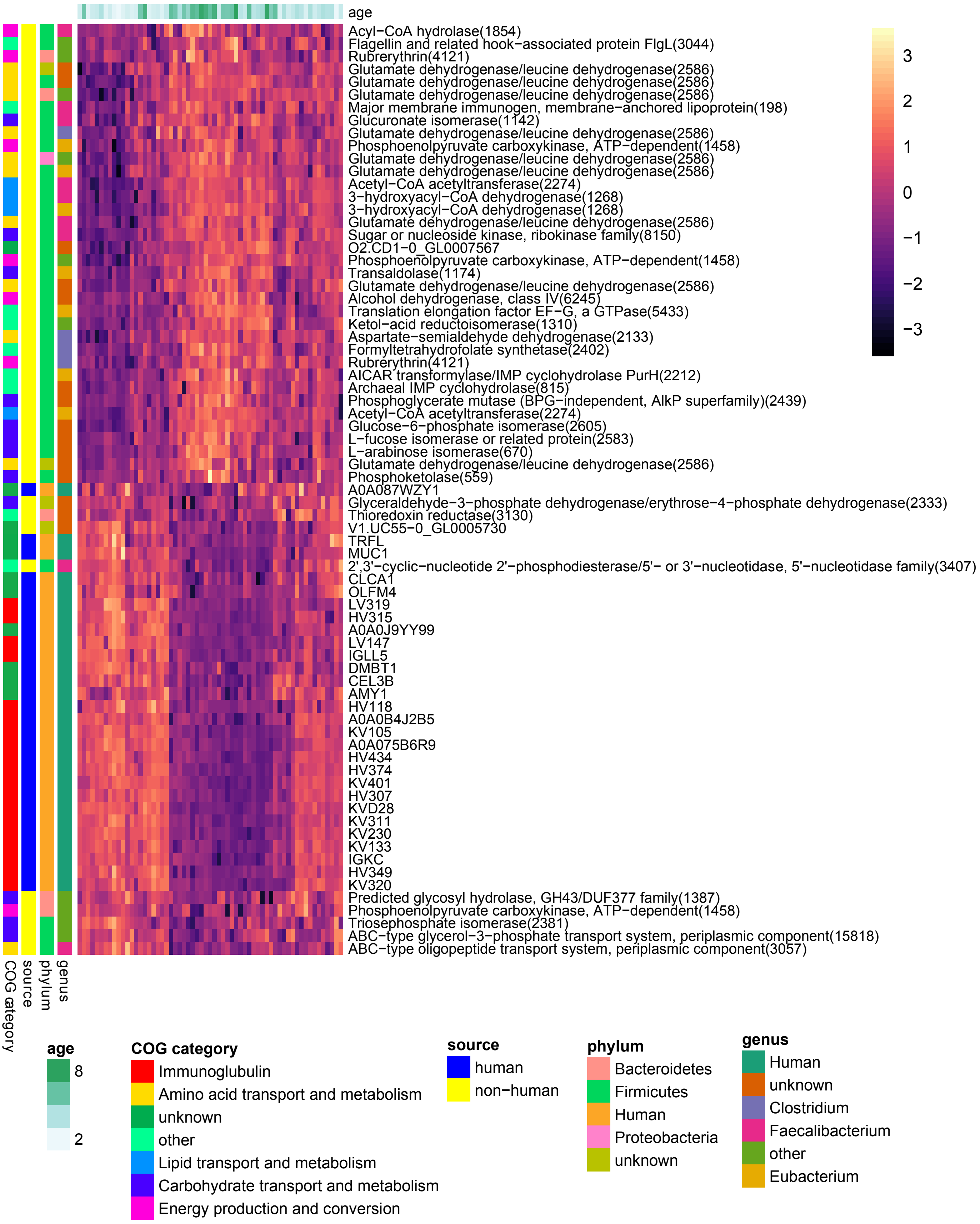

Supplementary Figure 3: Stool proteins associated with age. Linear mixed models identified 45 microbial and 28 human proteins associated with age, represented as a heatmap grouped using non-hierarchal clustering. Taxonomic and functional characteristics of the proteins are indicated by the color in the legend represented as a heatmap grouped using non-hierarchal clustering.

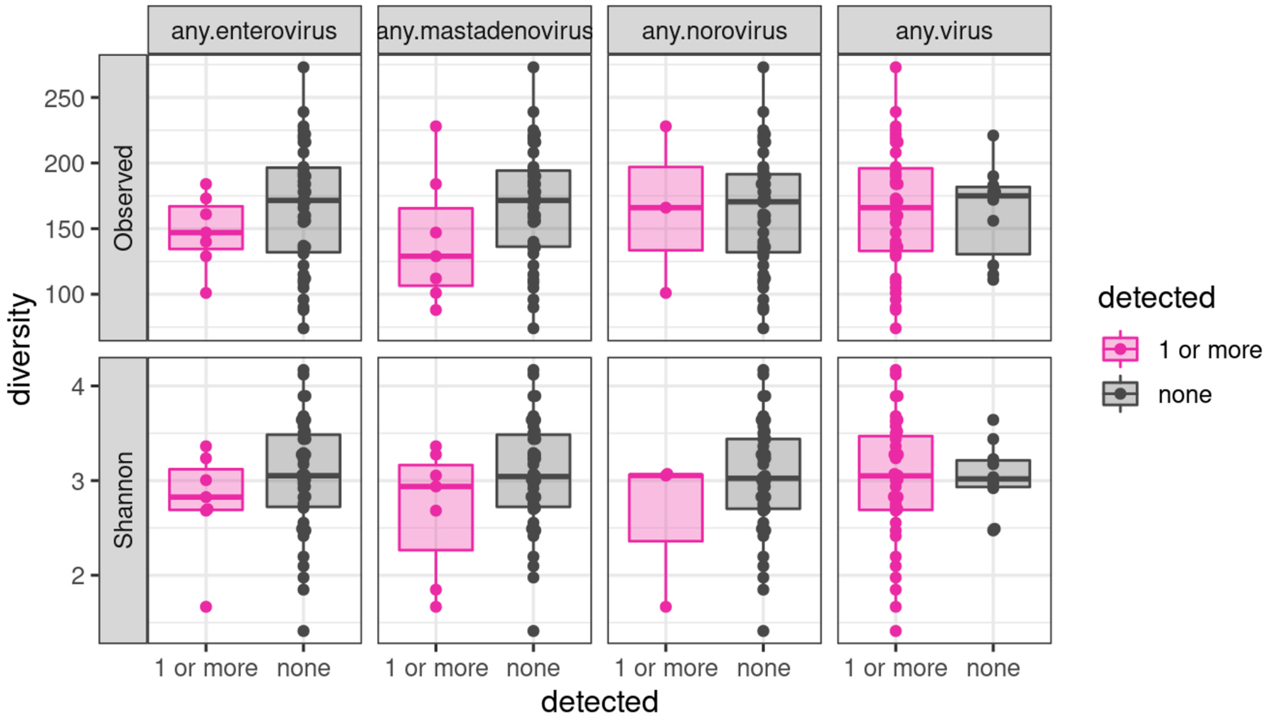

Supplementary Figure 4: Microbial diversity is not altered in the presence of virus. The Observed number of OTUs and the Shannon index tend to be lower in samples which have an infection, but these differences are not significant when adjusted for age (all q>0.1).

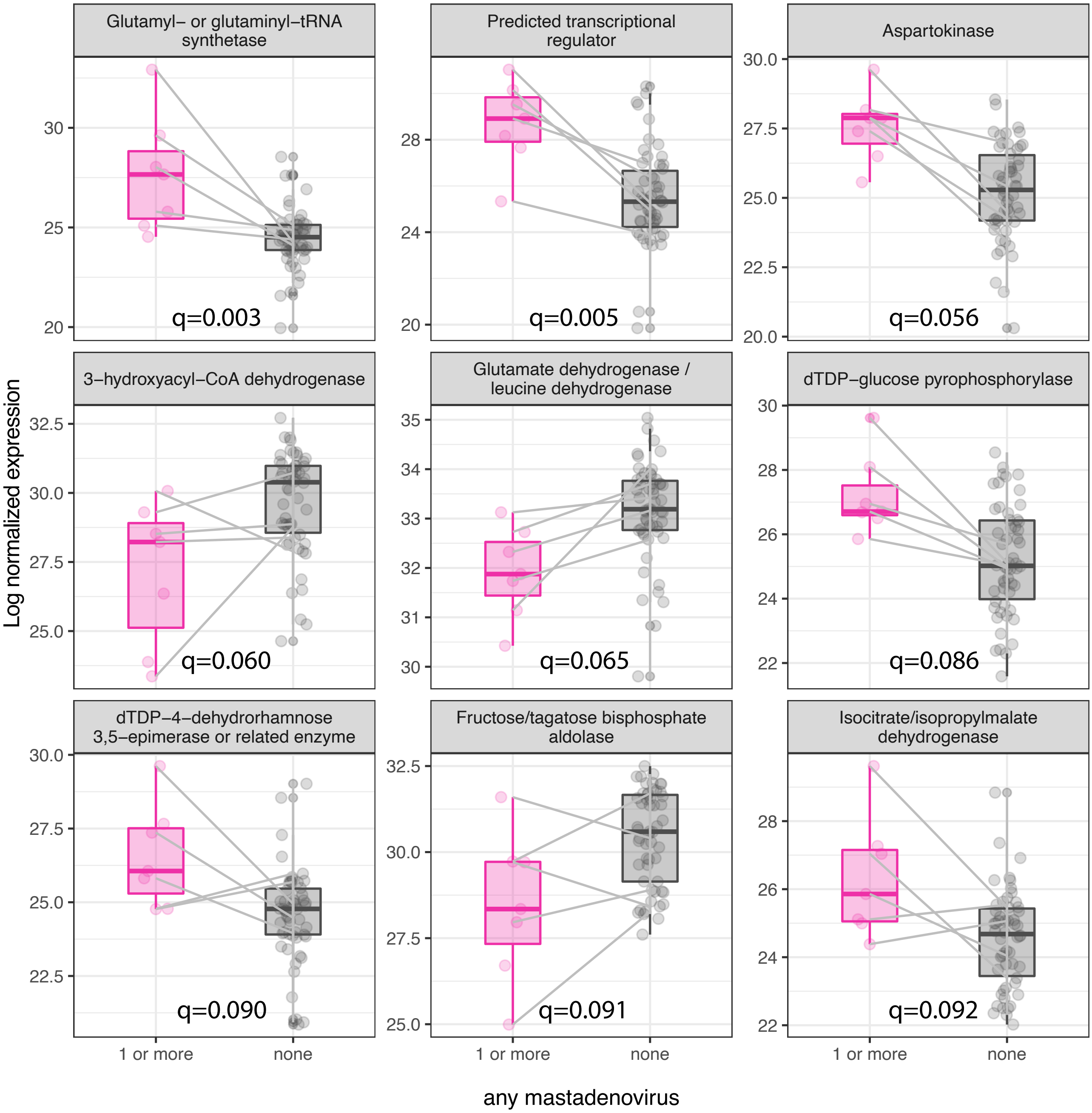

Supplementary Figure 5: Microbial functions associated with mastadenovirus. Proteins were aggregated by their Cluster of Orthologous Group (COG) assignments and evaluated for association with viral infection. Summed protein intensity from nine COGs shown are associated with the presence of mastadenovirus (q<0.1).

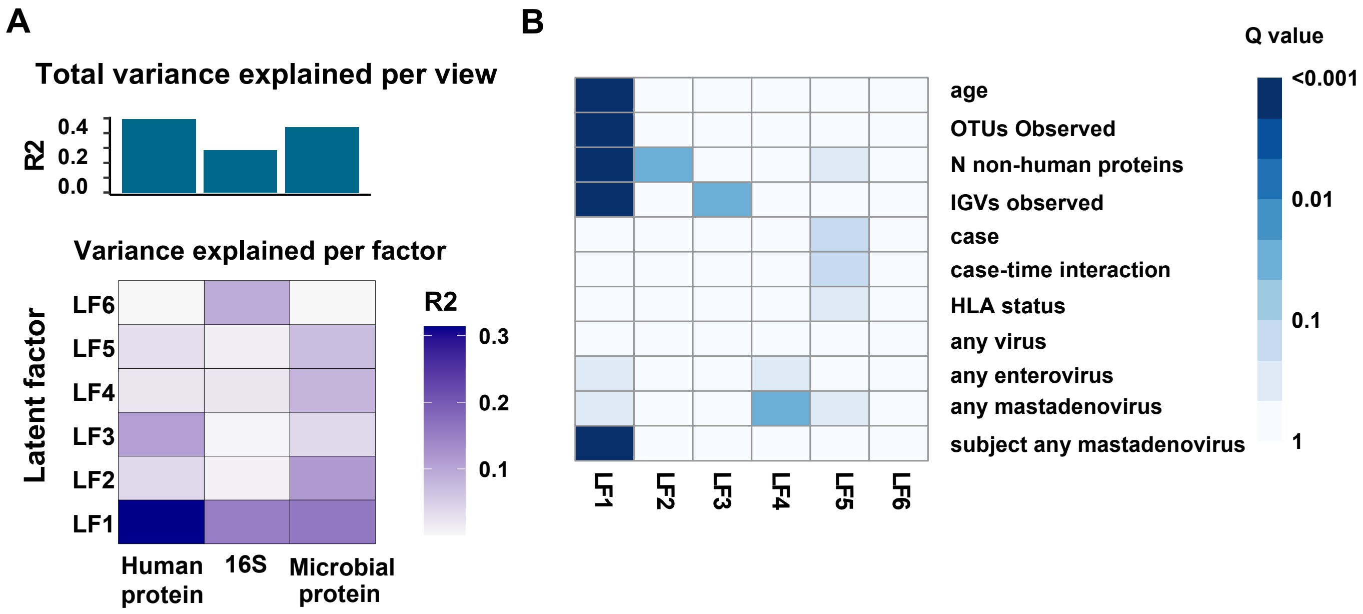

Supplementary Figure 6: **The virome explains little of the total variance following data integration**. MultiOmic Factor Analysis (MOFA) was used to integrate the virome, human proteome, microbial proteome, and 16S abundance estimates. **(A)** The total variance explained by each omic or "view", and **(B)** the variance explained for each latent factor.

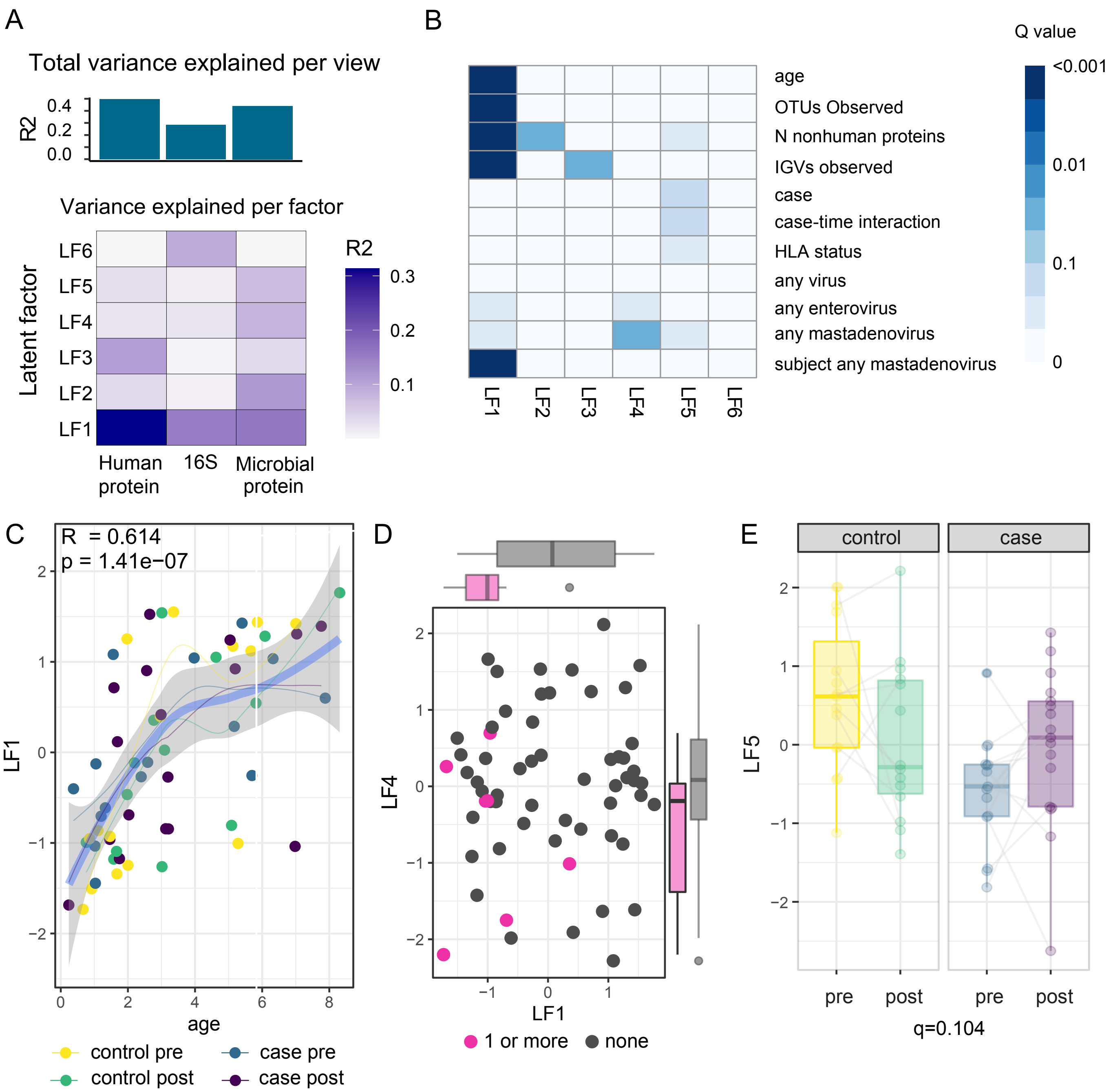

Supplementary Figure 7: MultiOmic Factor Analysis model 1 identifies latent factors associated with age, mastadenovirus and islet autoimmunity. (A) The total variance explained for each view and contribution to each latent factor (LF). (B) The association of the latent factors with other variables. Q-values from a linear mixed model adjusted for age, along with a representation of the weights for latent factor for those with an absolute weight >1 to any factor.
